# Long non-coding RNA RAD51-AS1 promotes the tumorigenesis of ovarian cancer by elevating EIF5A2 expression

**DOI:** 10.1007/s00432-024-05671-z

**Published:** 2024-04-07

**Authors:** Lu Zhao, Jia Huang, Wenting Liu, Xiaoyan Su, Bei Zhao, Xianggang Wang, Xiaoju He

**Affiliations:** 1 Department of Obstetrics and Gynecology, Second Affiliated Hospital of Nanchang University, No. 1 Minde Road, Nanchang, 330006 Jiangxi China; 2 Reproductive Health Department, Jiangxi Provincial Maternal and Child Health Hospital, Nanchang, Jiangxi China; 3 Pathology Department, Second Affiliated Hospital of Nanchang University, Nanchang, Jiangxi China; 4 Traditional Chinese Medicine Department, Duchang County People’s Hospital, Jiujiang, Jiangxi China

**Keywords:** Ovarian cancer, RAD51-AS1, EIF5A2, miR-140-3p

## Abstract

**Purpose:**

The present study aims to determine the molecular mechanism mediated by RAD51 antisense RNA 1 (RAD51-AS1) in ovarian cancer (OvCA).

**Methods:**

The data associated with RAD51-AS1 in OvCA were obtained from the Cancer Genome Atlas (TCGA) and the Gene Expression Omnibus (GEO) database. Relative expression of RAD51-AS1 was detected. Determination of cell proliferation, metastasis, and invasion was performed by cell counting, colony formation, would-healing, and transwell invasion assays. Protein levels were detected by western blotting. The molecular mechanism mediated by RAD51-AS1 was predicted by bioinformatics analysis and verified by dual-luciferase reporter assays. Subcutaneous tumorigenesis models were used to confirm the function of RAD51-AS1 in vivo.

**Results:**

Data from TCGA and GEO showed that RAD51-AS1 was associated with poor prognosis in OvCA patients and DNA repair, cell cycle, focal adhesion, and apoptosis in SKOV3.ip cells. High levels of RAD51-AS1 were detected in OvCA cells. Overexpressing RAD51-AS1 enhanced the proliferative, invading, and migratory capabilities of OvCA cells in vitro while silencing RAD51-AS1 exhibited the opposite effects. Mechanically, RAD51-AS1 elevated eukaryotic initiation factor 5A2 (EIF5A2) expression as a sponge for microRNA (miR)-140-3p. Finally, the role of RAD51-AS1 was verified by subcutaneous tumorigenesis models.

**Conclusion:**

RAD51-AS1 promoted OvCA progression by the regulation of the miR-140-3p/EIF5A2 axis, which illustrated the potential therapeutic target for OvCA.

**Supplementary Information:**

The online version contains supplementary material available at 10.1007/s00432-024-05671-z.

## Introduction

Ovarian cancer (OvCA) ranks eleventh and fifth among common cancers and related deaths in female patients, respectively (Morand et al. 2021). Approximately 140,000 women die from OvCA worldwide every year (Penny 2020). The main treatment method for patients with OvCA is to remove all macroscopic diseases through cytoreductive surgery (Feng et al. 2018; Ayhan and Akilli 2021). Approximately 80% of OvCA patients receive treatment regimens of cytoreductive surgery combined with adjuvant chemotherapy (Baci et al. 2020). However, about 70% of patients who adopt this treatment regimen relapse again, and recurrent patients typically have resistance to standard platinum chemotherapy (Nowak and Klink 2020). For patients in advanced stages, only about 29% of patients have a 5-year survival time (Siegel et al. 2019). More molecular changes in the population of OvCA patients have been identified, and exploring these abnormal molecules will have significant implications for exploring new diagnostic and therapeutic targets for OvCA patients (Guan and Lu 2018; Norouzi-Barough et al. 2018).

The largest group of non-coding RNAs (ncRNAs) produced in the genome is long non-coding RNAs (lncRNAs) (Robinson et al. 2020). The key feature of these transcripts is that they can not encode proteins and exceed 200 nucleotides in length (Bridges et al. 2021). Mechanically, lncRNAs exert their regulatory effects on various cancer-related cellular processes through communication with proteins, ncRNAs, mRNAs, and DNA by serving as different functional molecules, such as decoys and scaffolds (Ørom et al. 2010; Winkle et al. 2021). For instance, tumorigenic lncRNA on chromosome 1p13 could lead to the downregulation of some tumor suppressors by interacting with TPR in liver cancer (Yuan et al. 2022). LncRNA KCNQ1 opposite strand/antisense transcript 1 exerted a tumor-promoting function in OvCA by decreasing eukaryotic translation initiation factor 2B (EIF2B5) expression by recruiting DNA methyltransferases into EIF2B5 promoter (He et al. 2022a, b). In colorectal cancer, NOTCH1-associated lncRNA in T cell acute lymphoblastic leukemia 1 strengthened the proliferative and migrating capacities of the tumor cells by sponging microRNA (miR)-574-5p (Ye et al. 2022). LncRNA RAD51 antisense RNA 1 (RAD51-AS1) located on 15q15.1 has been reported to improve the effectiveness of chemo/radiotherapy for cancer cells in liver cancer (Chen et al. 2018a, b) and repress cancer cell growth in colorectal cancer (Chen et al. 2018a, b; Li et al. 2021a, b). On the contrary, RAD51-AS1 could promote the growth of tumor cells in breast cancer (Gazy et al. 2015) and OvCA (Zhang et al. 2017), but to date still little is known about molecular mechanisms by which RAD51-AS modulates OvCA progression.

The eukaryotic initiation factor 5A2 (EIF5A2) gene located on chromosome 3q26 was first discovered in a primary OvCA cell line (Guan et al. 2001). Significant upregulation of EIF5A2 was verified in prostate cancer, and interference with EIF5A2 in PC-3 M IE8 cells decreased cell growth and lung metastasis in mouse models. The ubiquitination of EIF5A2 mediated by HERC3 repressed tumor cell invasion and migration in colorectal cancer (Zhang et al. 2022). Inhibition of EIF5A2 lowered hypoxia-induced cisplatin resistance by repressing hypoxia-induced autophagy in lung cancer (Xu et al. 2022). More importantly, the oncogenic role of EIF5A2 was also confirmed in OvCA (Wang et al. 2021; Zhao et al. 2021). A previous study uncovered the downregulation of EIF5A2 in RAD51-AS1-inhibiting SKOV3.ip cells (Zhang et al. 2017). However, whether RAD51-AS can regulate OvCA progression through EIF5A2 remain unknown.

Here, we aimed to investigate the molecular mechanisms related to RAD51-AS1 and EIF5A2 in OvCA.

## Experimental procedures

### Online bioinformatics analysis and prediction

The clinical data related to diagnosis and prognosis for RAD51-AS1 in OvCA was download from The Cancer Genome Atlas (TCGA; https://portal.gdc.cancer.gov/). The OvCA microarray gene profiling datasets (GMS2367520, GMS2367521, GMS2367522, GMS2367523, GMS2367524 and GMS2367525) (Zhang et al. 2017) in this research were downloaded from the Gene Expression Omnibus (GEO) database (https://www.ncbi.nlm.nih.gov/) and analyzed using “limma” package.

### Cell incubation

Human ovarian surface epithelial cells (HOSEpiC) (#7310, Zhongqiao Xinzhou Biotechnology Co., Ltd, Shanghai, China) were cultured using an ovarian epithelial cell culture medium (Zhongqiao Xinzhou Biotechnology Co., Ltd). The medium used for SKOV3 cells (HTB-77™, ATCC, Manassas, VA, USA) was McCoy’s 5A encompassing 10% fetal bovine serum (FBS, Thermo, Waltham, MD, USA). The medium used for OVCAR3 cells (HTB-161™, ATCC) was RPMI-1640 Medium (Thermo) encompassing 20% FBS and Insulin (0.01 mg/mL, Sigma, St. Louis, MO, USA). All media encompassed 100 U/mL penicillin (Sigma) and 100 μg/mL streptomycin (Sigma). Cell incubation was carried out at standard cell culture conditions (37˚C and 5% carbon dioxide).

### Reverse transcription quantitative polymerase chain reaction (RT-qPCR)

The RNeasy Mini Kit (Qiagen, Valencia, CA, USA) was used for the isolation of total RNA from cells. For the detection of RAD51-AS1 and EIF5A2, the complementary (c) DNA was obtained using the First Strand cDNA Synthesis Kit (Takara, Japan). For miR-140-3p detection, total RNA underwent reverse transcription for cDNA production in the light of the instructions provided by the miRNA First Strand cDNA Synthesis kit (Sangon, Shanghai, China). The reaction was about qPCR being run using real-time fluorescence quantitative PCR instruments (ABI 7500, Applied Biosystems, Foster City, CA, USA) with SYBR Green Master Mix (Takara). All primers were provided in Table 1. Relative expression of RAD51-AS1, EIF5A2 and miR-140-3p was calculated using the 2^−ΔΔCt^ method by normalizing to glyceraldehyde-3-phosphate dehydrogenase (GAPDH) or U6 expression.

**Table 1:** Sequences of PCR primers used in this study

RAD51-AS1	Forward (5ʹ-3ʹ)	TACTGCCGAAACAAACCACA
	Reverse (5ʹ-3ʹ)	CCACGACTCCCAAGAGGTAA
EIF5A2	Forward (5ʹ-3ʹ)	GCTTCCAGCACTTACCCTATG
	Reverse (5ʹ-3ʹ)	ACTATTTTGCATGGTCGTCCTTT
miR-140-3p	Forward (5ʹ-3ʹ)	ACACTCCAGCTGGGAGGCGGGGCGCCGCGGGA
	Reverse (5ʹ-3ʹ)	CTCAACTGGTGTCGTGGA
GAPDH	Forward (5ʹ-3ʹ)	TGTTCGTCATGGGTGTGAAC
	Reverse (5ʹ-3ʹ)	ATGGCATGGACTGTGGTCAT
U6	Forward (5ʹ-3ʹ)	CTCGCTTCGGCAGCACA
	Reverse (5ʹ-3ʹ)	AACGCTTCACGAATTTGCGT

### Cell transfection

For pcDNA-RAD51-AS1 (RAD51-AS1) and pcDNA-EIF5A2 (EIF5A2) plasmids, the full-length cDNA sequences of RAD51-AS1 and EIF5A2 were cloned into the pcDNA3.1 empty vector (Thermo, Waltham, MA, USA). The FuGENE HD transfection reagent (Promega, Madison, WI, USA) was used for plasmid transfection and the stable cells were selected with G418 (0.5 mg/mL) (Sigma). For RAD51-AS1 knockdown, two short hairpins (sh) RNAs targeting RAD51-AS1 (sh1-RAD51-AS1 and sh2-RAD51-AS1) were inserted into the pLKO.1 vector (Addgene, Cambridge, MA, USA). HEK293 cells were co-transfected with a recombinant sh1-RAD51-AS1/sh2-RAD51-AS1 plasmid and lentivirus packing plasmids (pMD2G and psPAX2, Addgene) to produce lentivirus particles using Lipofectamine 3000 reagent (Thermo). OvCA cells were infected with lentivirus particles under polybrene (8 mg/mL, Sigma) and selected with puromycin (5 μg/mL, Thermo). MiR-140-3p inhibitor, NC inhibitor, miR-140-3p mimic and NC mimic were synthesized by (GenePharma, Shanghai, China) and transfected by Lipofectamine 3000 reagent (Thermo).

### Cell counting kit-8 (CCK-8) assay

Trypsin-digested transfected OvCA cells (about 1.5 × 10^3^) were inoculated in a 96-well plate. After 1 or 2 days of cell adhesion, the supernatant was removed, followed by the addition of CCK-8 solution (10 µL, Beyotime, Shanghai, China) and complete culture medium (100 µL). The absorbance (450 nm) was evaluated using an absorbance reader (BioTek, Winooski, VT, USA) after incubation at 37 °C for 2 h.

### Colony formation assay

Approximately 1 × 10^5^/well transfected OvCA cells were seeded on 6-well plates and incubated for 10–15 days at 37 °C. Visible colonies were fixed with 4% paraformaldehyde (Sigma) followed by staining with 0.1% crystal violet (Beyotime). The colonies were imaged under a microscope (Olympus, Tokyo, Japan) and counted manually.

### Wound-healing assay

In short, transfected OvCA cells (1 × 10^5^/wells) were seeded in 6-well dishes. After reaching about 80–90% confluence, cells were scratched from the bottom of the wells with a sterile 200 uL pipette tip. Capturing images and measuring were performed at 0 and 24 h after the wound was created.

### Transwell invasion assay

The serum-free medium (500 μL) containing 1 × 10^5^ OvCA cells was added into the upper chamber (8.0 μm, Costar, Cambridge, MA, USA) pre-coated with Matrigel (BD Biosciences, San Jose, CA, USA). The DMEM medium containing 10% FBS was added into the bottom chamber. After 24 h of incubation, the cells in the bottom chamber were stained with 0.1% crystal violet (Beyotime). Counting of the number of invading cells was performed under a light microscope (Olympus).

### Western blotting

The radioimmunoprecipitation assay (RIPA) lysis buffer (Beyotime) was used to extract total protein from OvCA cells. Determination of protein concentration was conducted with a BCA Protein Assay Kit (Thermo). 40 μg protein samples were loaded onto fresh sodium dodecyl sulfate polyacrylamide gel electrophoresis gel (10%). After isolating, these bands were then electro-transferred onto a PVDF membrane (Millipore, Massachusetts, USA) and then blocked with 5% skimmed milk, followed by incubation with the following primary antibodies against EIF5A2 (ab126733, 1:5000, Abcam, Cambridge, MA, USA), proliferating cell nuclear antigen (PCNA) (AF1363, 1:1000, Beyotime), Ki67(AF1738, 1:1000, Beyotime), cyclin D1 (ab16663, 1:200, Abcam), cyclin-dependent kinases 4 (CDK4) (ab108357, 1:1000, Abcam), cleaved caspase 3 (c-caspase 3) (ab32042, 1:500, Abcam), Bax (AF1270, 1:1000, Beyotime), Bcl-2 (AF1225, 1:1000, Beyotime), (E-cadherin (ab40772, 1:10,000, Abcam), N-cadherin (ab76011, 1:1000, Abcam) and Vimentin (AF1975, 1:1000, Beyotime), or anti-GAPDH (ab181602, 1:10,000, Beyotime) at 4 °C overnight and then incubated with secondary antibodies for 1 h. The integrated optical density was quantified with Image J software (NIH, Bethesda, MD, USA) after detecting the protein bands using the ECL system (Thermo).

### Dual-luciferase reporter assay

For the evaluation of the relationship between RAD51-AS1 and EIF5A2, the EIF5A2 promoter region was inserted into a pGL3-basic luciferase vector (Promega). The fragments containing the binding sites for miR-140-3p in RAD51-AS1 or 3’UTR of EIF5A2 were cloned into pmirGLO vector separately and named RAD51-AS1 WT and EIF5A2 WT. Generation of the mutated plasmids (RAD51-AS1 MUT and EIF5A2 MUT) was achieved by mutating the putative binding sites in RAD51-AS1 or 3’UTR of EIF5A2. OvCA cells were co-transfected with miR-140-3p inhibitor or NC inhibitor and a luciferase reporter. Forty-eight hours post-transfection, all luciferase activities were then assessed by the Dual-Luciferase Reporter Assay System (Promega).

### Subcutaneous tumorigenesis model

The in vivo experiments were approved and manipulated based on protocols approved by the Animal Care Committee of the Second Affiliated Hospital of Nanchang University. Eighteen female BALB/c nude mice (Animal Experimental Science Center of Nanchang University, Nanchang, China) were randomly divided into three groups: control (*n* = 6), sh-NC (*n* = 6), and sh2-RAD51-AS1 (*n* = 6). All mice were fed at 25 ± 2 °C (45–50% constant humidity). SKOV3 cells or SKOV3 cells with sh-NC or sh2-RAD51-AS1 (1 × 10^7^) were subcutaneously injected into the right hindlimb of each mouse. Tumor volume was measured once a week by measuring their length (*l*) and width (*w*) and calculating the volume (*V*) as follows: *V* = *l* × *w*^2^/2. Four weeks post-injection, all mice were euthanized, followed by measurement and weighing of excised tumors. Paraffin-embedded tumor samples were subjected to immunohistochemistry (IHC) staining to detect EIF5A2-positive cells.

### Statistical analysis

All experiments in vitro were performed at least triplicate biological replicates. Data analysis was implemented by GraphPad Prism 8 (GraphPad Software, USA) and presented as the mean ± SEM (standard error of the mean). For the comparison between the two groups, the difference between normal distribution variables was calculated using an independent Student’s *t*-test, and the difference between non-normal distribution variables was calculated using Mann–Whitney *U*-test. Comparison of multiple groups was made by analysis of variance with Tukey’s correction. Significance was defined as any statistical result that resulted in a *p*-value less than 0.05.

## Results

### 3.1 RAD51-AS1 was associated with poor prognosis in OvCA patients and DNA repair, cell cycle, focal adhesion, and apoptosis in SKOV3.ip cells

**Fig. 1:**
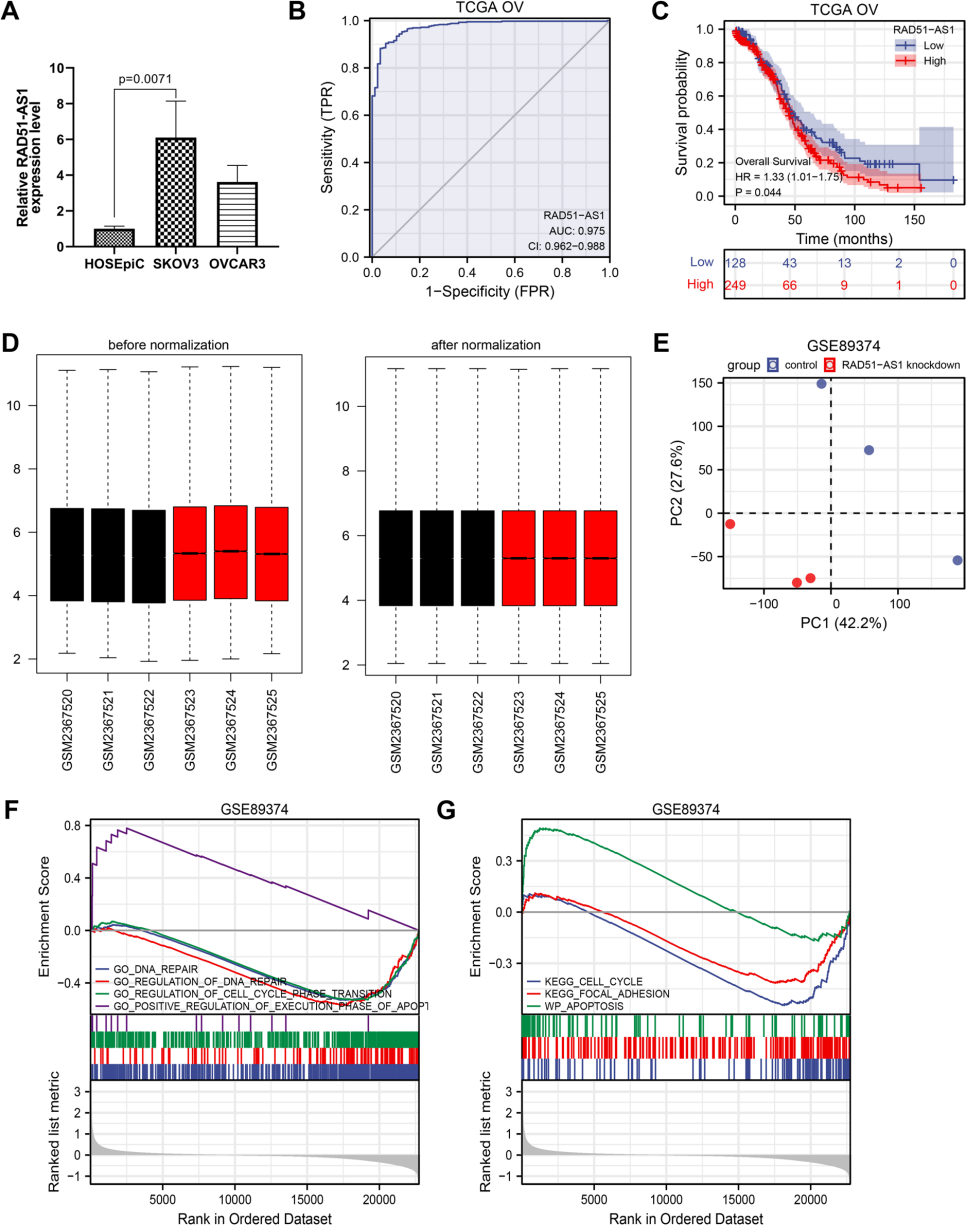
RAD51-AS1 was associated with poor prognosis in OvCA patients and DNA repair, cell cycle, focal adhesion, and apoptosis in SKOV3.ip cells. **A** Relative expression of RAD51-AS1 in OvCA cell lines detected by RT-qPCR (*p* = 0.0071 vs. HOSEpiC). **B** ROvCA curve analysis for the diagnostic performance of RAD51-AS1 in OvCA samples. **C** Kaplan–Meier survival curves showed the survival differences in OvCA patients with high or low RAD51-AS1 expression. **D** Box-plots about RAD51-AS1 expression in 6 datasets (GMS2367520, GMS2367521, GMS2367522, GMS2367523, GMS2367524 and GMS2367525) before and after normalization. **E** Principal component analysis of SKOV3.ip cells and RAD51-AS1-knockdown SKOV3.ip cells in GSE89374 data. **F** and **G** GSEA-enrichment plot of 4 biological processes and 3 pathways

Relative RAD51-AS1 expression in OvCA cell lines was first estimated by RT-qPCR. Higher levels of RAD51-AS1 in SKOV3 and OVCAR3 cell lines were observed in comparison with the HOSEpiC cell line (Fig. 1A). In the TCGA cohort, the area under the curve (AUC) for OvCA diagnosis was 0.975 (CI: 0.962–0.988) in the analysis of the receiver operating characteristics (ROC) curve of RAD51-AS1, suggesting that RAD51-AS1 might serve as an improved diagnostic biomarker for OvCA (Fig. 1B). Furthermore, OvCA patients with low RAD51-AS1 expression survived longer in the TCGA cohort (Fig. 1C).

For the bioinformatics analysis on the GSE89374 dataset, the box plot results showed that the expression levels in different datasets were not on the same horizontal line, so we performed normalization to remove batch effects (Fig. 1D). To distinguish significant differences between normal cells and RAD51-AS1-knockdown cells, principal component analysis was performed to reduce dimensionality and assess the independence of each group. The results showed that there were significant differences between SKOV3.ip cells and RAD51-AS1-knockdown SKOV3.ip cells (Fig. 1E). Moreover, the gene set enrichment analysis (GSEA) indicated that RAD51-AS1 was involved in four biologic processes (DNA repair, regulation of DNA repair, regulation of cell cycle phage transition, and positive regulation of execution phase of APOP1) and 3 pathways (cell cycle, focal adhesion and apoptosis) (Fig. 1F andG). These bioinformatics analyses collectively suggested that high RAD51-AS1 expression was associated with poor prognosis in OvCA patients, and RAD51-AS1 might be involved in DNA repair, cell cycle, focal adhesion, and apoptosis of OvCA cells.

### RAD51-AS1 facilitated OvCA cell proliferation, migration, invasion and EMT

**Fig. 2:**
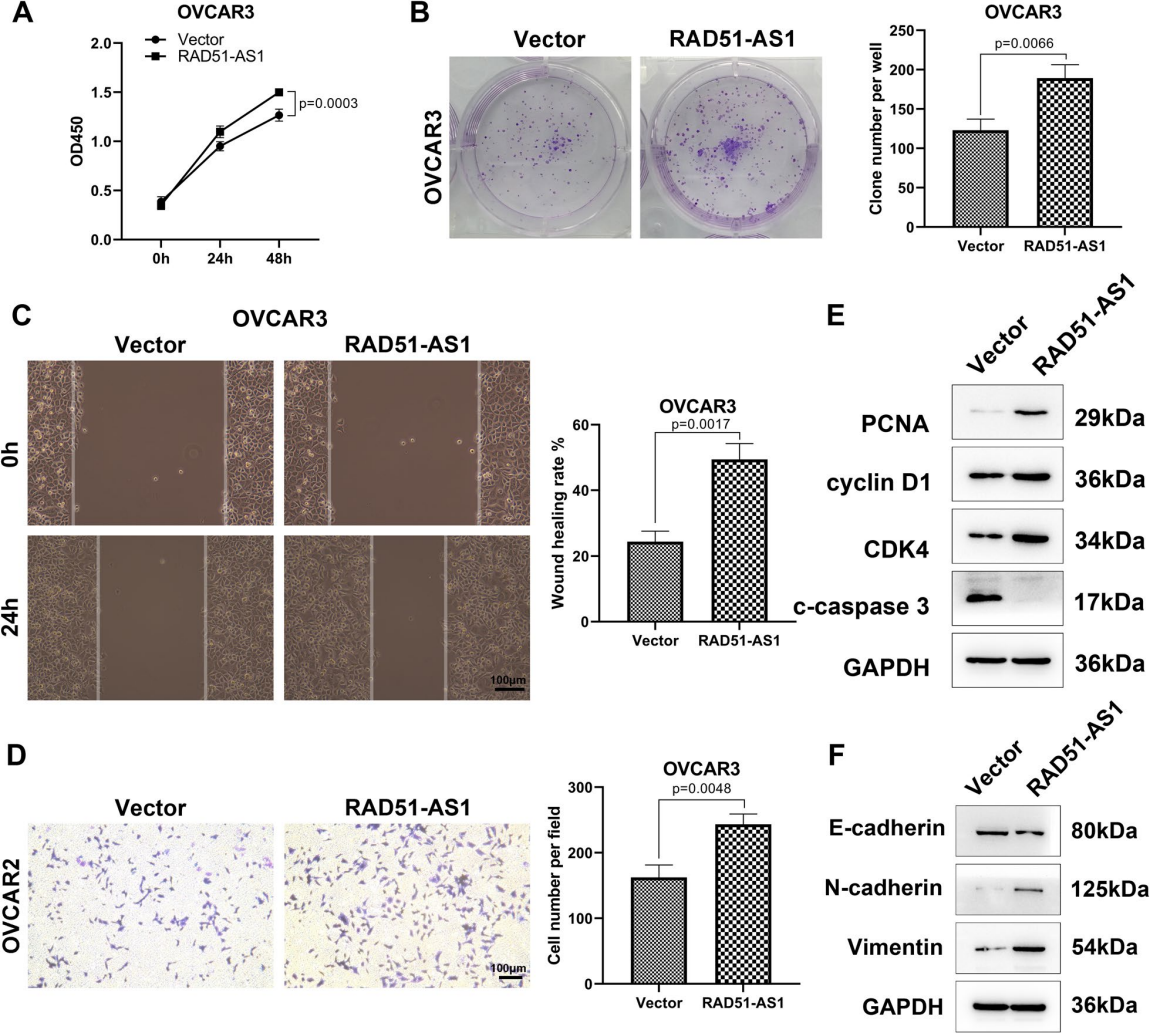
RAD51-AS1 markedly elevated OVCAR3 cell proliferation, migration, invasion and EMT. **A** The growth curve of CCK-8 assay for OVCAR3 cells with overexpression of RAD51-AS1 (*p* = 0.0003 vs. Vector). **B**–**D** Representative images and quantitative results of colony formation, wound-healing and transwell invasion assays for OVCAR3 cells with overexpression of RAD51-AS1 (*p* = 0.0066, *p* = 0.0017 and *p* = 0.0048 vs. Vector). **E** and **F** Western blotting was conducted to detect PCNA, cyclin D1, CDK4, c-caspase 3, E-cadherin N-cadherin and Vimentin protein levels in RAD51-AS1-overexpressing OVCAR3 cells and control cells

To determine the biological function of RAD51-AS1 in OvCA, we established RAD51-AS1-overexpressing OVCAR3 cells (Figure S1A). As expected, RAD51-AS1 overexpression strengthened the proliferative and colony formation abilities of OVCAR3 cells (Fig. 2A and B). Furthermore, the migratory and invading capacities of OVCAR3 cells were also markedly enhanced in RAD51-AS1-overexpressing OVCAR3 cells (Fig. 2C and D). To corroborate the above changes, some molecular markers related to cell proliferation, cell cycle, apoptosis, and EMT were detected. Western blotting results demonstrated a significant upregulation of PCNA, cyclin D1, CDK4, N-cadherin, and Vimentin in RAD51-AS1-overexpressing cells, whereas c-caspase 3 and E-cadherin protein levels were significantly downregulated (Fig. 2E and F). Collectively, RAD51-AS1 promoted OVCAR3 cell proliferation, migration, invasion, and EMT.

### Inhibition of RAD51-AS1 diminished SKOV3 cell proliferation, migration, invasion, and EMT

**Fig. 3:**
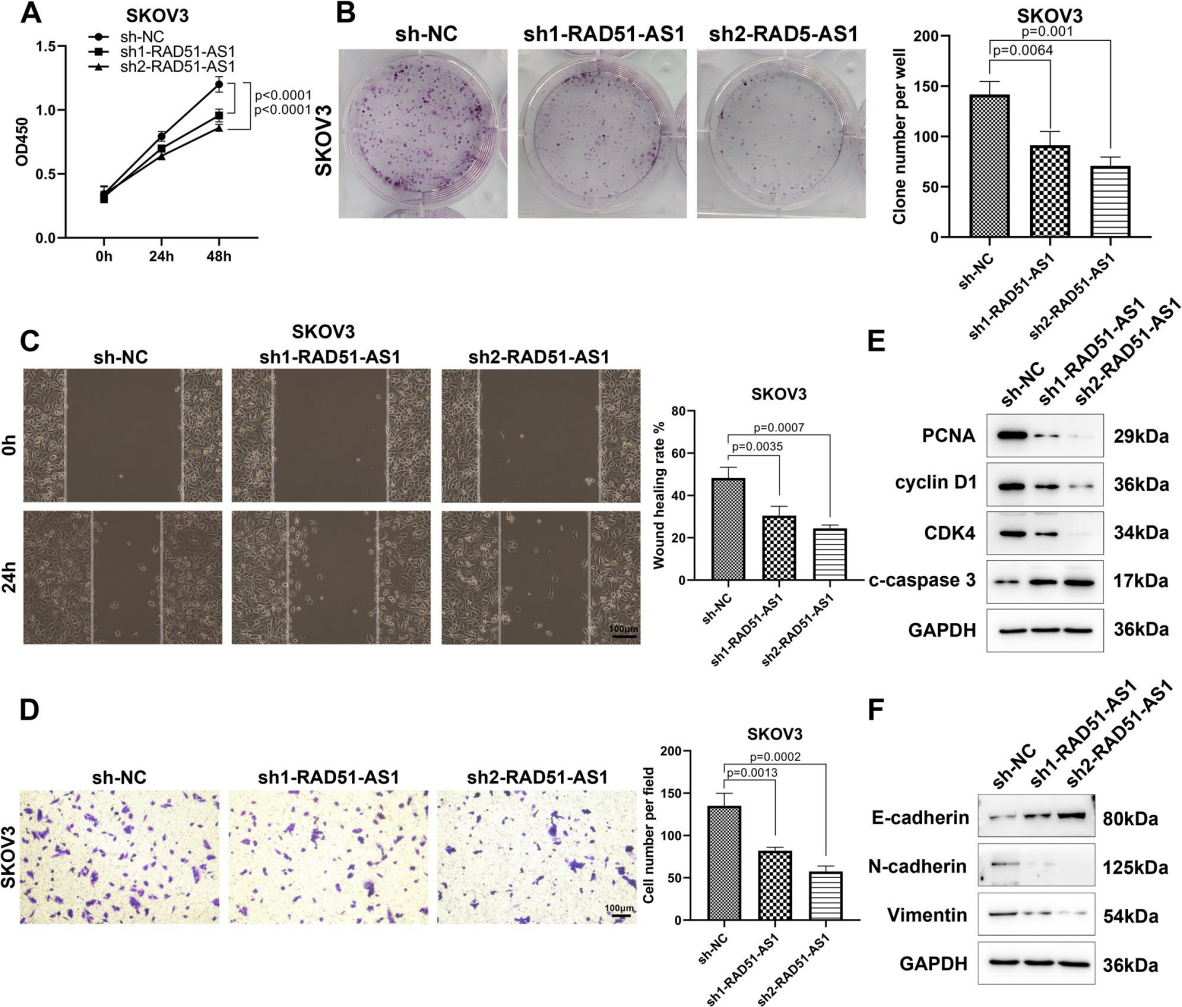
RAD51-AS1 silencing inhibited SKOV3 cell proliferation, migration, invasion and EMT. **A** The CCK-8 assay analyzed the alteration of proliferative ability following the knockdown of RAD51-AS1 in SKOV3 cells (*p* < 0.0001 vs. sh-NC). **B–D** Representative images and quantitative results of colony formation (*p* = 0.001 and *p* = 0.0064 vs. sh-NC), wound-healing (*p* = 0.0007 and *p* = 0.0035 vs. sh-NC) and transwell invasion assays (*p* = 0.0002 and *p* = 0.0013 vs. sh-NC) for SKOV3 cells with knockdown of RAD51-AS1. **E** and **F** Detection of PCNA, cyclin D1, CDK4, c-caspase 3, E-cadherin N-cadherin and Vimentin protein levels in RAD51-AS1-inhibited SKOV3 cells and control cells were done by western blotting

Next, we established RAD51-AS1-knockdown SKOV3 cells for the assessment of the biological function of RAD51-AS1, and the knockdown efficiency was exhibited in Figure S1B. Contrary to the overexpression of RAD51-AS1, RAD51-AS1 silencing markedly decreased the proliferation, colony formation, migration and invasion of SKOV3 cells (Fig. 3A–D). Consistently, PCNA, cyclin D1, CDK4, N-cadherin, and Vimentin protein levels in SKOV3 cells were downregulated, whereas c-caspase 3 and E-cadherin protein levels were upregulated after knocking down RAD51-AS1 (Fig. 2E and F). These results supported that RAD51-AS1 might be required for SKOV3 cell proliferation, migration, invasion, and EMT.

### RAD51-AS1 mediated SKOV3 cell proliferation, migration, invasion, and EMT via regulation of EIF5A2 expression

**Fig. 4:**
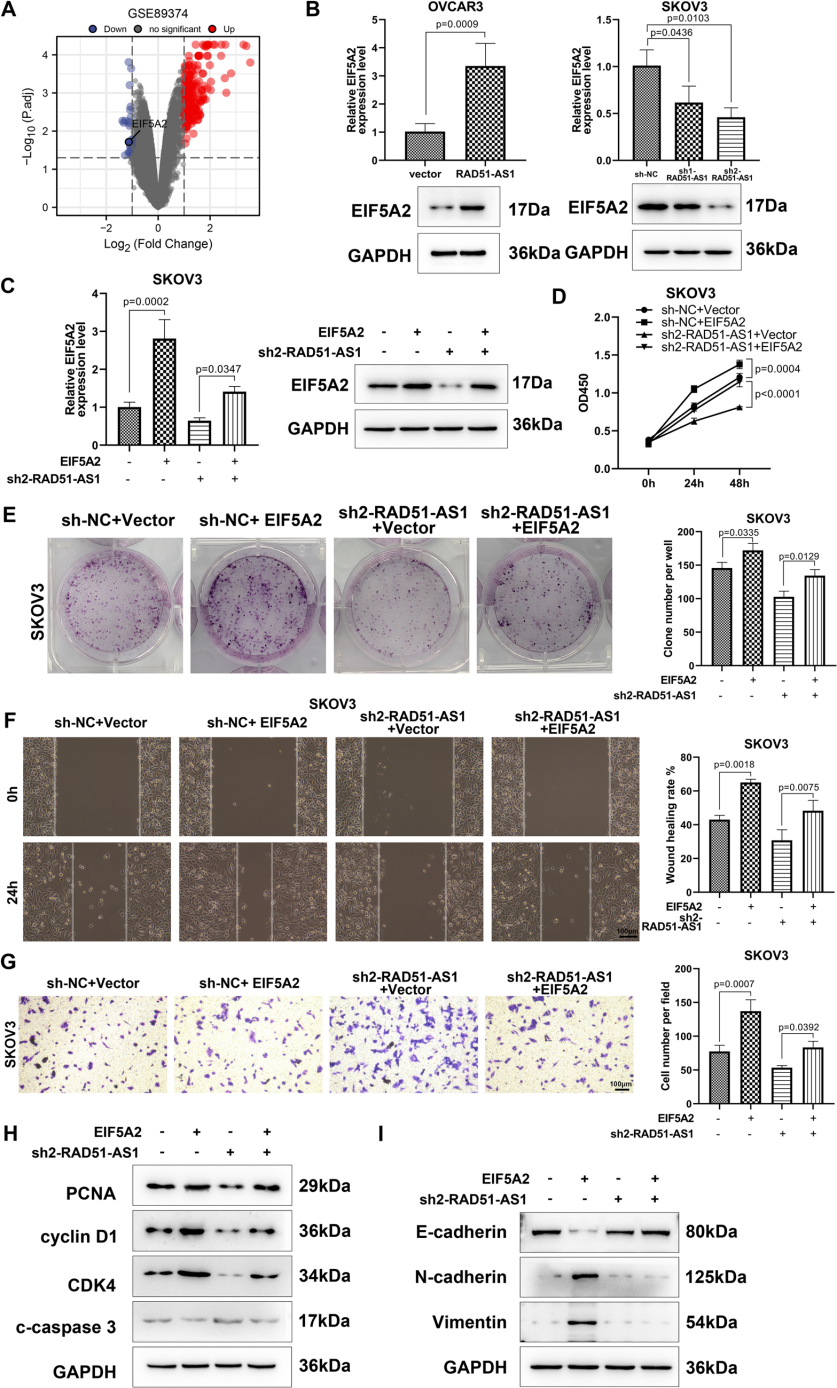
RAD51-AS1 promoted the malignant behaviors of OvCA cells by elevating EIF5A2 expression. **A** Volcano plot of differentially expressed genes in RAD51-AS1-knockdown SKOV3.ip cells (GSE89374; *p* < 0.05, |fold change|> 2). **B** Detection of EIF5A2 protein levels was done in RAD51-AS1-overexpressing OVCAR3 cells (*p* = 0.0009 vs. Vector) and RAD51-AS1-inhibiting SKOV3 cells (*p* = 0.0103 vs. sh-NC). **C** Western blotting assessed EIF5A2 protein levels in SKOV3 cells transfected with sh-NC + EIF5A2 (*p* = 0.0002 vs. sh-NC + Vector) or sh2-RAD51-AS1 + EIF5A2 (*p* = 0.0347 vs. sh2-RAD51-AS1 + Vector). **D** Cell colony formation ability was determined in SKOV3 cells transfected with sh-NC + EIF5A2 or sh2-RAD51-AS1 + EIF5A2 (*p* = 0.0335 vs. sh-NC + Vector; *p* = 0.0129 vs. sh2-RAD51-AS1 + Vector). **E** Cell migration was assessed in the above cell lines by wound-healing assays (*p* = 0.0018 vs. sh-NC + Vector; *p* = 0.0075 vs. sh2-RAD51-AS1 + Vector). **F** Cell invasion was determined in the above cell lines (*p* = 0.0007 vs. sh-NC + Vector; *p* = 0.0392 vs. sh2-RAD51-AS1 + Vector). **H** and **I** Protein levels of PCNA, cyclin D1, CDK4, c-caspase 3, E-cadherin N-cadherin, and Vimentin were measured in the above cell lines

To explore the molecular mechanism by which RAD51-AS1 promotes OvCA progression, we screened for significant differential expressed genes between OvCA cells with and without RAD51-AS1 knockdown and plotted the results into a volcano plot (*p* < 0.05, |fold change|> 2) based on GSE89374 dataset. Among them, EIF5A2 with a significant downregulation differential change attracted our attention (Fig. 4A). Western blotting showed that EIF5A2 protein levels were markedly upregulated in RAD51-AS1-overexpressing OVCAR3 cells and downregulated in RAD51-AS1-inhibiting SKOV3 cells, implying that RAD51-AS1 positively regulated EIF5A2 expression in OvCA cells (Fig. 4B). Rescue experiments were then conducted in SKOV3 cells to determine whether RAD51-AS1 promotes OvCA progression through EIF5A2. We constructed an EIF5A2 overexpression plasmid in SKOV3 cells and observed that the downregulation of EIF5A2 caused by RAD51-AS1 silencing was weakened following the EIF5A2 introduction (Fig. 4C). Meanwhile, EIF5A2 promoted SKOV3 cell proliferation, colony formation, migration, and invasion, and the introduction of EIF5A2 disminished the inhibitory effects of RAD51-AS1 silencing on the malignant behaviors of SKOV3 cells (Fig. 4D–G). Correspondingly, the changes in protein levels of PCNA, cyclin D1, CDK4, c-caspase 3, E-cadherin N-cadherin, and Vimentin mediated by EIF5A2 knockdown were weakened after overexpression of EIF5A2 (Fig. 4G and H). Together, RAD51-AS1 modulated the malignant behaviors of OvCA cells through EIF5A2.

### RAD51-AS1 modulated EIF5A2 expression by interacting with miR-140-3p

**Fig. 5:**
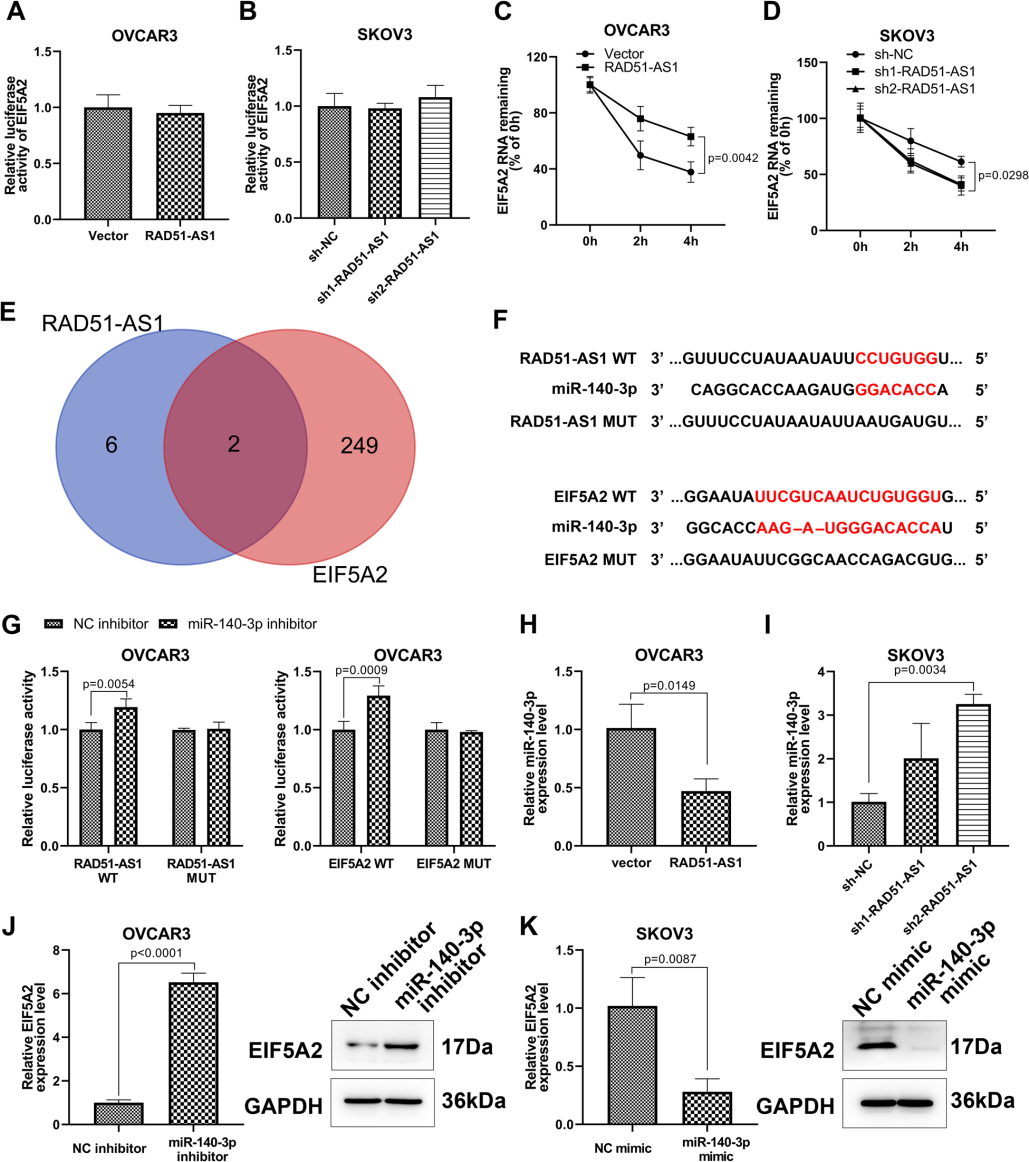
RAD51-AS1 regulated EIF5A2 expression by sponging miR-140-3p. **A** and **B** Dual-luciferase reporter assays detected the relative luciferase activity of EIF5A2 in RAD51-AS1-overexpressing OVCAR3 cells and RAD51-AS1-knockdown SKOV3 cells. **B** and **C** RT-qPCR detection of EIF5A2 mRNA in RAD51-AS1-overexpressing OVCAR3 cells (*p* = 0.0042 vs. Vector) and RAD51-AS1-knockdown SKOV3 cells (*p* = 0.0298 vs. sh-NC) incubated with actinomycin D. **C** A schematic drawing of the screening procedure for candidate miRNAs (miR-154-5p and miR-140-3p). **D** A schematic drawing of the binding sites with miR-182-5p in RAD51-AS1 and EIF5A2, as well as their mutation sites. **E** Dual-luciferase reporter assays detected the relative luciferase activity in OVCAR3 cells transfected with RAD51-AS1 WT + miR-140-3p inhibitor (*p* = 0.0054 vs. RAD51-AS1 WT + NC inhibitor), RAD51-AS1 MUT + miR-140-3p inhibitor (ns vs. RAD51-AS1 MUT + NC inhibitor), EIF5A2 WT + miR-140-3p inhibitor (*p* = 0.0009 vs. EIF5A2 WT + NC inhibitor) or EIF5A2 MUT + miR-140-3p inhibitor (ns vs. EIF5A2 MUT + NC inhibitor). **G** and **H** RT-qPCR was used to analyze miR-140-3p expression in RAD51-AS1-overexpressing OVCAR3 cells (*p* = 0.0149 vs. Vector) and RAD51-AS1-knockdown SKOV3 cells (*p* = 0.0034 vs. sh-NC). **J** and **K** Relative mRNA and protein levels of EIF5A2 in OVCAR3 cells with knockdown of miR-140-3p (*p* < 0.0001 vs. NC inhibitor) and SKOV3 cells with overexpression of miR-140-3p (*p* = 0.0087 vs. NC mimic)

To evaluate whether RAD51-AS1 directly interacts with EIF5A2, we constructed a dual-luciferase reporter plasmid containing the EIF5A2 promoter. As shown in Fig. 5A and B, either overexpression or knockdown of RAD51-AS1 did not have a significant effect on the fluorescence activity of the plasmid, implying that EIF5A2 expression was indirectly regulated by RAD51-AS1. SKOV3 cells with silenced RAD51-AS1 or OVCAR3 cells with overexpression of RAD51-AS1 were incubated with actinomycin D and then subjected to analyzing the expression of EIF5A2. RT-qPCR results manifested that RAD51-AS1 upregulation prolonged the half-life of EIF5A2 mRNA while RAD51-AS1 silencing reduced the half-life of EIF5A2 mRNA (Fig. 5C and D). Accumulative evidence has indicated that lncRNAs can function as miRNA sponges. Therefore, we predicted miRNAs bound to RAD51-AS1 or EIF5A2 using ENCORI. Two miRNAs (miR-154-5p and miR-140-3p) that interact with them collectively were obtained through Venn analysis, and miR-140-3p downregulated in OvCA was selected for further analysis (Fig. 5E). Interestingly, miR-140-3p inhibitor markedly elevated the luciferase activity in OVCAR3 cells of the RAD51-AS1 WT and EIF5A2 WT groups, but there were no changes in the RAD51-AS1-MUT and EIF5A2 MUT groups (Fig. 5F and G). We also observed the downregulation of miR-140-3p in RAD51-AS1-overexpressing OVCAR3 cells and the upregulation of miR-140-3p in RAD51-AS1-knockdown SKOV3 cells (Fig. 5H and I). In addition, the miR-140-3p inhibitor increased EIF5A2 expression at mRNA and protein levels in OVCAR3 cells (Fig. 5J). On the contrary, the miR-140-3p mimic resulted in a significant reduction of EIF5A2 in SKOV3 cells (Fig. 5K). All results manifested that RAD51-AS1 functioned as a miR-140-3p sponge and regulated EIF5A2 expression by sponging miR-140-3p.

### RAD51-AS1 silencing decreased SKOV3 cell growth in vivo

**Fig. 6:**
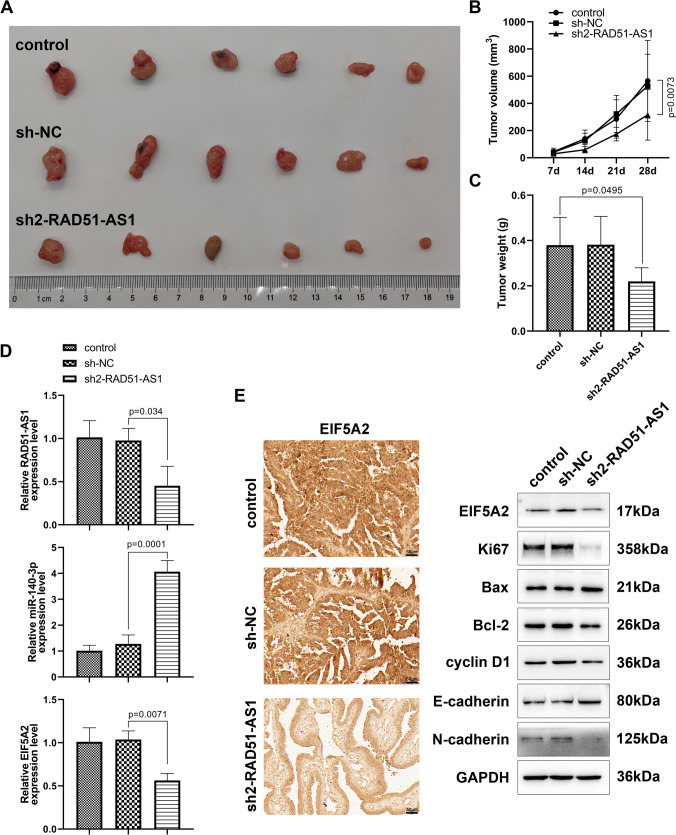
Inhibition of RAD51-AS1 lowered SKOV3 cell growth in vivo. **A** Images of the xenograft tumors formed in nude mice of the control, sh-NC and sh2-RAD51-groups. **B** The growth curve of the xenograft tumors in the sh2-RAD51-AS1 group (*p* = 0.0073 vs. sh-NC). **C** The weight of the xenograft tumors isolated from nude mice in the sh2-RAD51-AS1 group (*p* = 0.0495 vs. sh-NC). **D** Relative expression levels of RAD51-AS1 (*p* = 0.034 vs. sh-NC), miR-140-3p (*p* = 0.0001 vs. sh-NC) and EIF5A2 mRNA (*p* = 0.0071 vs. sh-NC) in the xenograft tumors of the sh2-RAD51-AS1 group were detected. **E** Representative images of IHC staining of EIF5A2 in the xenograft tumors derived from nude mice; Relative protein levels of EIF5A2, Ki67, Bax, Bcl-2, cyclin D1, N-cadherin and E-cadherin in the xenograft tumors isolated from nude mice were detected by western blotting

Subcutaneous tumorigenesis models were then established by subcutaneous injection of SKOV3 cells or SKOV3 cells infected with lentivirus-mediated sh2-RAD51-AS1 or sh-NC. The tumor size, growth rate, and weight of tumors derived from cells with RAD51-AS1 knockdown were significantly lower than those of the control and sh-NC groups (Fig. 6A–C). Tumors derived from cells with RAD51-AS1 knockdown showed lower levels of RAD51-AS1 and EIF5A2 mRNA than those in the control and sh-NC groups, but miR-140-3p expression was higher (Fig. 6D). IHC staining showed that the EIF5A2 index in tumors derived from cells with RAD51-AS1 knockdown was significantly lower than that in the other 2 groups (Fig. 6E). What is more, the diminished protein levels of EIF5A2, Ki67, Bcl-2, cyclin D1, and N-cadherin were observed in tumors of the sh2-RAD51-AS1 group in comparison to the other 2 groups, whereas Bax and E-cadherin protein levels were elevated (Fig. 6E). In summary, RAD51-AS1 contributed to the growth of SKOV3 cells in nude mice.

## Discussion

Molecular targeted therapy has become another tumor treatment method following surgery and radio/chemotherapy, with the continuous development of biotechnology and molecular biology (Li et al. 2021a, b). There is strong evidence suggesting the importance of lncRNAs in many biological processes. Recent reports have uncovered that abnormally expressed lncRNAs in OvCA play an important role in tumor progression (Wang et al. 2019; Braga et al. 2020). Therefore, exploring the molecular mechanisms associated with lncRNAs in the development of OvCA is of great significance for finding new molecular targets and screening suitable targeted therapeutic drugs.

Previous studies have reported the different functions of RAD51-AS1 in different tumors. The elevation of RAD51-AS1 was uncovered to repress cell invasion, glycolysis process, and migration by mediating NDRG2 expression via sequestering miR-29b/c-3p, leading to the reduction of colorectal cancer progression (Li et al. 2021a, b). Similarly, the upregulation of RAD51-AS1 enhanced cell sensitivity to chemotherapy by binding to RAD51 mRNA and repressing its translation in liver cancer (Chen et al. 2018a, b; Chen et al. 2018a, b). Oppositely, RAD51-AS1 was overexpressed in OvCA samples and correlated with poor survival in epithelial OvCA patients, and p53 was repressed by RAD51-AS1, leading to the promotion of cell proliferation (Zhang et al. 2017). In our study, bioinformatics analysis showed the diagnostic and prognostic value of RAD51-AS1 for OvCA and suggested that RAD51-AS1 was related to DNA repair, cell cycle, focal adhesion, and apoptosis in OvCA cells. Our data showed the overexpression of RAD51-AS1 in OvCA cells, and further function analyses showed the contributory effect of RAD51-AS1 on cell malignancy in OvCA cells in vitro, including proliferative, invasive, migratory, and EMT, as well as tumor growth in mouse models, supporting the oncogenic role of RAD51-AS1 in OvCA.

Based on bioinformatics analysis of differentially expressed genes in OvCA cells caused by RAD51-AS1 knockdown, we discovered that RAD51-AS1 might regulate EIF5A2 expression in OvCA cells. EIF5A2 has been revealed as a new independent predictor of outcomes in OvCA patients (Yang et al. 2009). The contributory effect of EIF5A2 on OvCA cell stemness was caused by its mediation of the E2F1/KLF4 pathway (Wang et al. 2021). In addition, EIF5A2 enhanced tumor growth by facilitating the EMT process via the TGF-β pathway (Zhao et al. 2021). Here, further experimental results confirmed the negative regulation of EIF5A2 in OvCA cells by RAD51-AS1. Cell biological experiments further showed that EIF5A2 overexpression blocked the inhibiting effect of RAD51-AS1 silencing on OvCA cell proliferation, invasion, migration, and EMT, indicating that RAD51-AS1 modulated the malignancy of OvCA cells through EIF5A2.

For exploring the regulatory mechanism between RAD51-AS1 and EIF5A2, the luciferase reporter plasmid with EIF5A2 promoter did not show significant differences in OvCA cells with overexpression or knockdown of RAD51-AS1, suggesting that RAD51-AS1 could not directly bind and interact with EIF5A2. Much evidence suggests that lncRNAs act as competitive endogenous RNAs by competitively sponging and sequestering miRs, thereby eliminating miRs-mediated inhibition of their target mRNAs and indirectly regulating the expression of mRNAs (Tay et al. 2014; Zhou et al. 2022). MiRs are very powerful genetic regulators, as evidenced by the fact that a single miR can interact with a broad spectrum of target genes and direct entire cellular pathways (Diener et al. 2022). Herein, miR-140-3p was predicted to interact with RAD51-AS1 and EIF5A2 jointly by ENCORI. The down-regulation and tumor inhibitory effect of miR-140-3p has been confirmed in a broad spectrum of cancers, such as bladder cancer (Yang et al. 2022), prostate cancer (He et al. 2022a, b), lung cancer (Huang et al. 2019), breast cancer (Dou et al. 2021) and gastric cancer (You et al. 2023). In OvCA, lncRNA AC005224.4-mediated upregulation of SNAI2 elevated cancer cell invasion and migration by sponging miR-140-3p (Xiong et al. 2023). Moreover, linc00852 sequestered miR-140-3p to elevate AGTR1 expression, ultimately contributing to the invading and proliferative capacities of OvCA cells (Qiao et al. 2021). More interestingly, we illustrated that RAD51-AS1 had a negative regulation on miR-140-3p and EIF5A2 was a miR-140-3p target by dual-luciferase reporter assays, RT-qPCR and western blotting. Our research results proposed a regulatory model in which high RAD51-AS1 expression sponged miR-140-3p to elevate EIF5A2 expression. Unfortunately, the risk model construction in the research was mainly based on the TCGA (OvCA cohort), so it is best to obtain sufficient clinical samples and data to verify the diagnostic and prognostic value of RAD51-AS1 in OvCA. What’s more, whether RAD51-AS1 plays a role in OvCA through other functions still needs to be explored, which is also worth exploring in future.

In summary, this work identified the oncogenic role of RAD51-AS1 in OvCA. Furthermore, RAD51-AS1 interacted with miR-140-3p, leading to the upregulation of EIF5A2, with the outcome of promoting tumor growth in OvCA. The research might be beneficial to further elucidate OvCA pathogenesis and provide new ideas for clinical treatment.

### Supplementary Information

Below is the link to the electronic supplementary material.

Figure S1. The expression of RAD51-AS1 in OvCA cells. (A) RT-qPCR was used to analyze the expression of RAD51-AS1 in OVCAR3 cells with overexpression of RAD51-AS1 (p = 0.0489 vs. vector). (B) RT-qPCR was used to analyze the expression of RAD51-AS1 in SKOV3 cells with knockdown of RAD51-AS1 (p = 0.0056 vs. sh-NC) (TIF 288KB)

## Data Availability

The datasets generated during and/or analyzed during the current study are available from the corresponding author on reasonable request.
