# Supplementary figures and images for: Long non-coding RNA RAD51-AS1 promotes the tumorigenesis of ovarian cancer by elevating EIF5A2 expression
Source: J Cancer Res Clin Oncol. 2024 Apr 7;150(4):179. doi: 10.1007/s00432-024-05671-z (PMC10999386; doi:10.1007/s00432-024-05671-z)

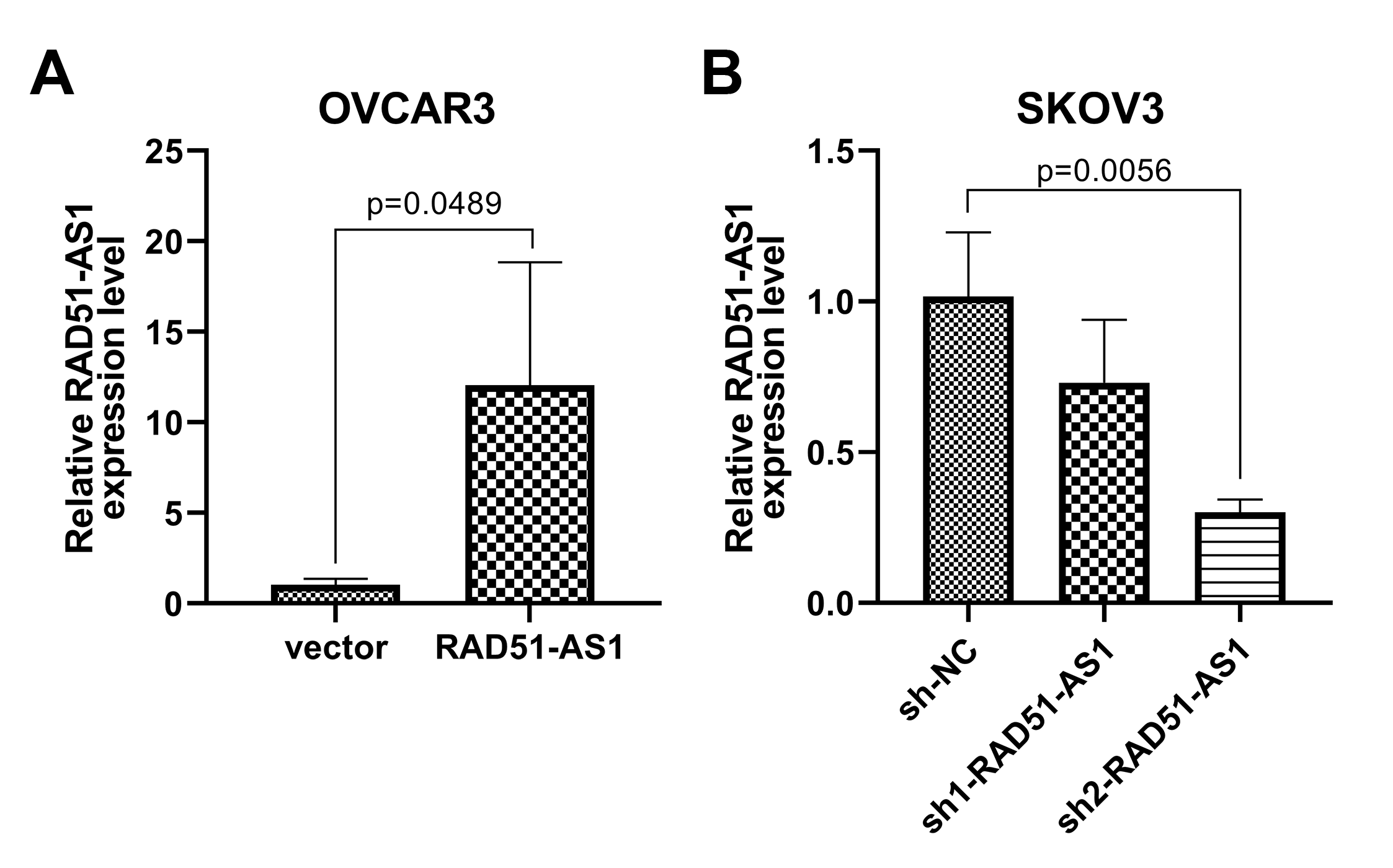

Supplement: Supplementary file 1 — Figure S1. The expression of RAD51-AS1 in OvCA cells. (A) RT-qPCR was used to analyze the expression of RAD51-AS1 in OVCAR3 cells with overexpression of RAD51-AS1 (p = 0.0489 vs. vector). (B) RT-qPCR was used to analyze the expression of RAD51-AS1 in SKOV3 cells with knockdown of RAD51-AS1 (p = 0.0056 vs. sh-NC) (TIF 288KB) [file 432_2024_5671_MOESM1_ESM.tif]
